# Cardiovascular adjustments during experimentally induced retraction and locomotion in the invasive terrestrial snail Cornu aspersum (Müller, 1774)

**DOI:** 10.1371/journal.pone.0354962

**Published:** 2026-07-31

**Authors:** Edgar Alejandro Pardo-Sarmiento, Juan Pablo Hernández, Natalia Buitrago-Ricaurte, Andre J. Riveros

**Affiliations:** 1 Departamento de Biología, Escuela de Ciencias e Ingeniería, Universidad del Rosario, Bogotá, Colombia; 2 Escuela de Medicina y Ciencias de la Salud, Universidad del Rosario, Bogotá, Colombia; 3 Department of Neuroscience, School of Science, University of Arizona, Tucson, Arizona. United States of America; Annamalai University, INDIA

## Abstract

The coupled function of physiology and behavior is crucial for generating survival responses. Generally, the cardiovascular system undergoes rapid adjustments during scape behavior to increase oxygen delivery to muscle tissues. In contrast, passive antipredator responses such as retraction in snails may impose mechanical constraints on the circulatory system. Here, we evaluated the cardiovascular response underlying the scape response (through induced retraction) and the physical activity in the invasive land snail *Cornu aspersum*. We quantified heart rate and heart rate variability in adult snails using laser optocardiography under the two conditions. We found that heart rate increased from retracted to the moving state and was accompanied by changes in heart rate variability and reduction of cardiac irregularities. Also, we found that locomotion intensity and body size did not influence these cardiac parameters. Our results suggest that metabolic demands, neural regulation and mechanical configuration collectively alter heart rate and heart rate variability. Thus, cardiovascular function is strongly dependent on behaviorally induced mechanical shifts in the circulatory system and on a “fight-or-flight”-like response.

## Introduction

Physiological and behavioral flexibility are cornerstones for animal survival in changing environments [1,2]. During the fight-or-flight response, vertebrates rapidly modify heart function parameters, including cardiac output (a conjugate of stroke volume and heart rate) and blood pressure, to maintain higher metabolic demands for the enhanced activity [3,4]. Similarly, invertebrates adjust cardiovascular responses during escaping, despite a generally lower metabolism [5]. For instance, an increased heart’s stroke volume precedes the fast propulsive scape of the cephalopod *Octopus vulgaris* [6]. Also, the marine gastropod *Clione limacina* enhances its heart rate during scape swimming [7].

As an alternative to these active responses, some animals exhibit passive behaviors when threatened. Gastropod snails, famously recognized by slow movements, primarily evolved defensive retractions into protective shells [8,9]. This strategy presumably requires less energy than active responses and might even lead to a depression of the cardiovascular function. Yet this strategy does not seem to preclude the evolution of active scaping in some species. For example, within the terrestrial genus *Karaftohelix*, *K. gainesi* and *K. selskii* are exceptions, exhibiting attacking behaviors [8]. This suggests the potential for flexible responses even in animals that typically display very slow locomotion. Does cardiovascular modulation underly these changes in physical activity resembling active responders?

Here, we aimed to tackle this question by comparing the heart activity of the terrestrial snail *Cornu aspersum* under the contrasting retracted and moving conditions. We hypothesized that the increase in energy needs of the muscular foot during movement would require higher hemolymph recirculation supported by higher heart rate and more regular activity. The reasons to select *C. aspersum* to test this hypothesis were threefold: 1. the locomotion, based on the wave-like contraction of the foot, is generally slow, yet may support movements of varying speeds [10] 2. this is an invasive species locally abundant that generally rely on retraction into the shell to avoid being preyed or injured [11,12] 3. the heart can be seen through the shell with proper lighting [13,14], opening the window to the development of an optimized system to record and analyze cardiovascular responses in detail.

As proxies for cardiovascular response, we primarily relied on heart rate. However, a custom-built laser optocardiograph coupled with detailed imaging analyses enabled us to track heart mechanical activity within a single visual plane. Thus, we were able to reconstruct and analyze the regularity of the heart activation via the oscillating contraction and relaxation of the cavities as well as the heart rate variability (HRV). HRV is a tool primarily used for the analysis of vertebrate heart function based on inferences about autonomic regulation. However, given its power to analyze regularities in waves, such as the observed in electrical and mechanical oscillations of heart function, HRV has been adapted for studies in invertebrates [15], despite divergent autonomic anatomical organization [16]. Lastly, we evaluated the effect of body size, a key morphological parameter with a broad impact across physiological systems. As snails grow shells for protection, we discriminated metabolic and non-metabolic tissue for our analyses. This is an important separation, because allometric growth of shells may lead to differences in energy allocated to movement [17], in turn affecting the cardiovascular response (see below).

Based on our experimental design, we predicted higher heart rate and enhanced regularity in the mechanical oscillations when comparing retracted *versus* moving states. Also, as we expected the snails to move at different speeds, we further predicted heart rate to increase with higher locomotor speed. Finally, as for the effect of body size, predictions are not easy to construct. While larger body size is generally associated with lower heart rate [18,19], we considered that this pattern might not hold in snails due to shell carrying. As land snails develop thicker shells for enhanced protection [20], their body size changes, leading to increased metabolic costs and modified anatomical organization [20–22]. Hence, the cardiovascular response may undergo compensatory adjustments to sustain the metabolic demands and mechanical constraints associated with larger size and the defense behaviors.

## Materials and methods

### Collection and maintenance of snails

We collected individuals of *Cornu aspersum* (Müller, 1774), an invasive land snail species, in urban gardens from Bogota, Colombia (2592 masl). Snails were collected from three urban gardens located in the western area of the city, consisting of small vegetation patches separated by approximately 7 m by paved ground (4°39′31.7″ N, 74°07′42.1″ W).

Snails were identified based on shell coloration and pattern according to standard descriptions [23], including the typical light-brown coloration and the presence of characteristic dark spiral banding consisting of five brown bands surrounding the shell.

Snails were maintained under laboratory conditions (Temperature: 21.3 ± 0.8°C, Relative humidity: 62 ± 5%; Fisherbrand™ Traceable™ Thermometer, Photoperiod: ~ 12h:12h light-dark cycle) in a glass aquarium (49x24x40 cm) with ventilation openings, a cup of humid soil and little aquarium rocks at the bottom. We fed the snails with Batavia lettuce *ad libitum* and white chalk as calcium supplement [24]. Before starting the experiments, the snails were allowed to acclimate for at least a month in the laboratory. The night before the experiments, we removed any food to avoid postprandial changes in heart rate activity [25,26].

### Characterization of body size

We measured body mass and shell size as proxies of body size. For body mass, we considered three measurements: total mass (hereafter Mass_T_: shell mass+soft tissue), shell mass and dry mass (dehydrated soft tissue). Mass_T_ was measured individually using an analytical scale (RADWAG, AS 220.R2 PLUS) prior to video-recording the heart under the two conditions (retracted and moving). After the experiments, the snails were sacrificed by immersion in liquid nitrogen for 60 s and dissected to separate the soft tissue from the shell. We then dehydrated the soft tissue at 60°C for 2 days and weighed the tissue. For shell size, we measured shell height and diameter [23,27] (digital caliper: Carrera Precision CP8812-T).

### Optocardiographic characterization of heart activity

**Fig 1:**
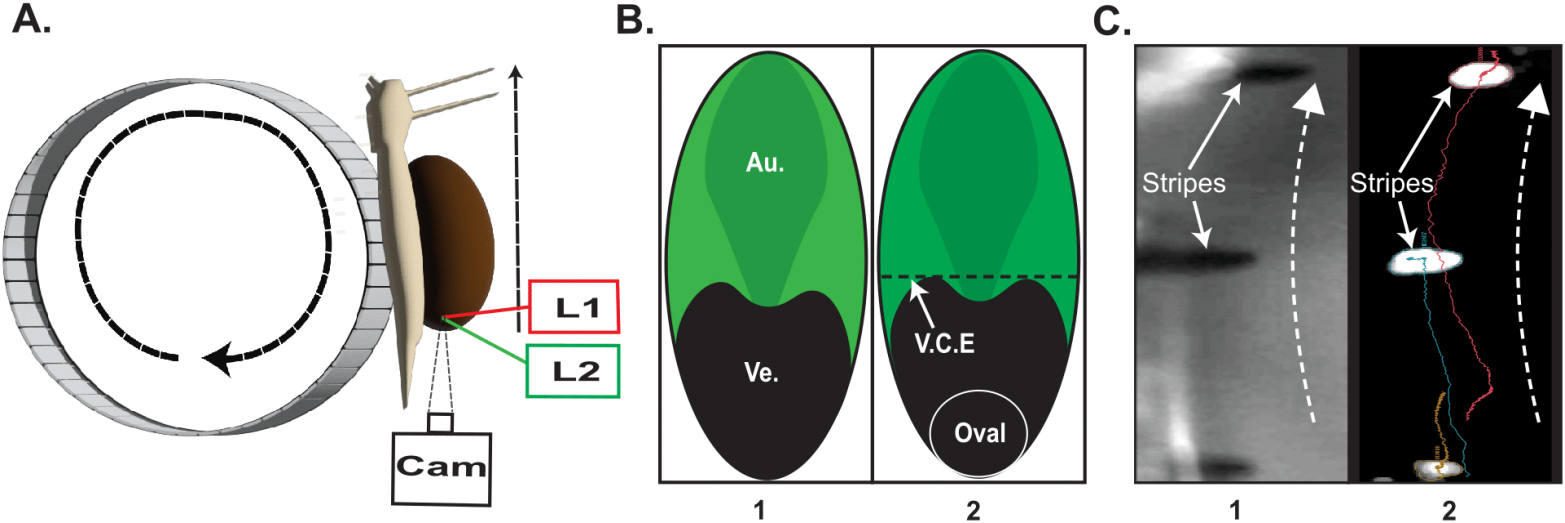
Video recording and imaging analysis of cardiac activity and locomotion. **A.** Optocardiographic setup. The snail is positioned upwards (black dashed arrows indicate direction) while the treadmill to the left rotates clockwise propulsed by the free movement of the snail (held in place). Two lasers (L1: Red; L2; Green) pointed to the shell to visualize cardiac movement, enabling continuous recording with a camera (Cam). **B1.** Schematic representation of the heart auricle (Au) and ventricle (Ve). The Ve was selected as the region of interest to track oscillations in contraction and relaxation using pixel-intensity changes **B2.** Schematic representation of the boundaries (V.C.E: ventricular contraction edge, black dashed line) and the regions considered for measuring pixel-intensity changes (oval/circle with a 50-pixel vertical axis/radius) used as proxies for ventricular contraction and expansion. **C.** Edited (1) and processed (2) images of the treadmill recordings used to calculate speed based on displacement of strips marked on surface. White arrow indicates treadmill movement direction (same as A).

For the visualization of cardiac movements, we held a snail with a built-in graduated clamp, ensuring that it remained in a vertical position with its body upwards (rubber tips were added to cushion the force and prevent the shells from breaking). Then, we directed a green (DHOM SLM DPSS, λ = 532 nm) and a red laser (DHOM CW DPSS, λ = 632 nm) above the shell (Fig 1A). The lasers were firmly placed with holders and screws, allowing precise adjustment. The convergence of the lasers allowed a direct visualization of the active heart through the intact shell.

For each snail, we recorded videos of the heart activity for 2 minutes (Canon 5D Mark II, 58 mm objective, magnification 4x and 10x). We edited the videos using *Adobe Premiere Pro*® (v. 25.2.3, Adobe Inc.) to remove fragments with noise and reduce file size, maintaining 80s of cardiac activity *per* snail. Then, we rotated the video to position the heart vertically (The ventricle is located below, and the atrium above; Fig 1B1). We used the circle crop tool to facilitate visualization of the heart. To optimize processing power, we split the video into four fragments (20 s each), enabling simultaneous visualization in the same window. We aligned the fragments using a grid (150 pt x 150 pt), carefully positioning every 20-second fragment (reviewing skew, skew axis, and the section scale) and maintaining an exact separation of 2 pt necessary to facilitate the use of a *Macro* routine (see below).

Then, we used *Fiji* [28] (ImageJ) to obtain optocardiogram curves describing contraction (systole) and relaxation (diastole) of the ventricle. Overall, we tracked the oscillation in pixel intensity within the ventricle following the observation that the contraction was associated with lower intensity (as the visualized area of the ventricle shrinked) and expansion with higher intensity (as the visualized area of ventricle enlarged). Specifically, we created a script in *Macro* language to acquire data for each video stack with a routine as follows: 1. transform the video from mp4 format to 8-bit, 2. enhance the contrast using *Lut*-*fire* tool. 3. if the pixels displayed an average intensity below 30, set at a minimum and maximum pixel intensity of −3 and 140, 4. trace a rectangle around the full area for each 20-second fragment and obtain its plot profile, 5. calculate the cumulative intensity changes through time for each vertical section (1-pixel width) within the rectangle. 6. determine the location with 85% of the highest change in intensity from the bottom (edge of the video; Fig 1B2: VCE) to the top (the zone displaying the expansion/contraction of the ventricle), 7. delimit a circular area (diameter: 50 pt) from the bottom of the fragment (this should establish an area for measuring pixel intensity within the ventricle, Fig 1B2), 8. if the circular area surpasses the 85% border, generate an oval extending from the left edge to the 85% limit (Fig 1B2).

After obtaining the oscillation of the average pixel intensity inside the oval/circular area, the data was processed in *R* (*R Core Team*, 2023, version 4.4.3) to exclude noise signals and find peaks and valleys of the signal (proxies of ventricle contraction and relaxation). First, we used the *sgolay* filter function to exclude noise. Second, we established a trend line with the *loess* function with a span of 0.3 using the libraries *patchwork* and *pracmq* to inspect peaks and valleys. We assigned a peak or valley area depending on the calculated derivative of pixel intensity (positive derivative indicated valley and negative derivative indicated peak). We selected the lower valley and the highest peaks for each 0.6 s aiming to avoid local peaks within the signal (minimum distance between consecutive peaks/valleys). Then, we converted frame sequences into time (one frame = 0.0333 s). For some pixel data, slight changes in the minimum distance parameter were necessary to avoid artifacts or skipped valleys. Together, these data were used to analyze heart rate and HRV parameters.

### Measuring heart rate (HR) and heart rate variability (HRV)

We analyzed the optocardiograms to determine oscillatory signals characterized by peaks (indicative of full ventricular relaxation) and valleys (corresponding to full ventricular contraction). We then used the systolic ventricular motion (valleys) to assess heart dynamics with HR and HRV parameters. The time differences between ventricular contractions during systole from one heartbeat to the following provided insights into the rhythm patterns of the cardiac cycle. SDNN and pNN100 were the time-domain metrics employed to estimate heart rate variability (S1 Table) as they are used to assess the overall short time fluctuations in NN intervals [29]. As resting heart rate is low in snails [14], we adjusted the scale of analysis for some HRV metrics. Thus, we used pNN100 instead of pNN50 [29], to ensure that the threshold for detecting beat-to-beat differences remained proportionate to the temporal scale of snail cardiac dynamics.

### Variation of cardiac activity in moving and retracted individuals

Snails were immobilized in a retracted state by wrapping the shell with parafilm (Parafilm® 7016−05), leaving a small opening to allow respiration through the pneumostome, the respiratory aperture that connects the lung cavity to the external environment. Immobilization was required to obtain stable and accurate heart recordings under the induced retracted condition. Without immobilization, extension of the body from the shell altered the degree of retraction, as well as heart position and light intensity, thereby reducing signal stability and introducing noise into the optical signal, which resulted in skipped beats or artifacts during parameter extraction. Cardiac recordings began 5 min after immobilization.

To assess cardiac activity during locomotion, snails were gently held in an upward orientation using graduated clamps on custom-made treadmill (Fig 1A). Because locomotion involves continuous body displacement that hinders stable optocardiographic recordings, individuals were positioned on the treadmill and lightly restrained to limit excessive movement while still allowing pedal waves to be observed as an indicator of muscular activity. This setup enabled reliable extraction of cardiac parameters under controlled conditions. The treadmill was built from a plastic circular band (diameter = 6.35 cm; width = 4 cm). The treadmill was held (PH6, PH4 Mini-Series Post Holders, Mph25 Thorlabs Inc.) such that we could adjust horizontal, vertical, and lateral axes. Then, we directed the lasers and proceeded to record the heart activity (see above).

Aiming to determine the speed of movement, we marked the plastic band with black stripes (every 5 mm) that allowed us to measure total displacement of the snails (Fig 1A). We conducted a separate video analysis for the plastic black stripes. This additional processing required further adjustments to brightness/contrast to enhance the correct visualization of the stripes and changed each section to grayscale with the tool tint (*Adobe Premiere Pro*®). Then, the position of visible stripes was obtained through *Fiji*. To do this, the videos were converted to 8-bit.mp4 format and each section of the recorded treadmill was individually selected. The brightness and contrast were set with a known distance of 5 mm between the two visible stripes. Then, we inverted the pixel intensity values to the stripes, creating a high intensity mark in a dark background (Fig 1C). Then, we applied a minimum and a maximum filter of two-pixel radius to enhance the definition of the stripes.

Under these pre-processing parameters, we applied a pre-trained Deep Learning Model of StarDist-2D for the automated detection and segmentation of the stripes [30,31] (Fig 1C). The segmented objects tracking was done using the *Trackmate* plugin [30] with the *LAP tracker* (Linear Assignment Problem) algorithm. During the detection step, the parameters related to object quality, area, and maximum intensity were adjusted within a restricted range according to the needs of each section and video, while manually verifying the reduction of incorrect detections throughout the recording. However, these restrictions were not applied too strictly to avoid excluding true stripe detections. Stripe detection was verified frame-by-frame to confirm that all stripes were correctly identified. For tracking, default parameters were used without modification, and the LAP tracker was employed. Detected stripe trajectories were then visually inspected using the TrackScheme interface, and only tracks that were consistently detected throughout the entire recording were retained. Incorrect detections, artifacts, and poorly linked tracks were manually removed. Only correctly detected treadmill stripes reviewed from the generated TrackMate XML file were used to estimate displacement and locomotion speed. The tracking results data (X position, frames, and ID) were exported as csv files for analysis in R.

The trajectories obtained were filtered and cleaned to avoid duplication and overlapping. To estimate the total displacement and speed, we used the spatial coordinates $x_{t}$ over time. We calculated the instantaneous speed (${Speed}_{ins}$) for each moment using a formula for one dimension displacement, establishing a frame duration of 0.0333 s per frame:


$$\begin{array}{c} {\;Speed}_{ins}=\frac{\left(x_{t}-x_{t-\text{1}}\right)}{\text{0}.\text{0}\text{3}\text{3}\text{3}\text{ s}} \end{array}$$ (1)

Then, we calculated the average speed (${Speed}_{av}$; cm min^-1^) for each snail:


$$\begin{array}{c} {\;Speed}_{av}=\frac{\text{1}}{n}\sum_{i=\text{1}}^{n}{Speed}_{instantaneous} \end{array}$$ (2)

Where n is the number of time points where ${Speed}_{ins}$ was calculated.

To validate the automated methodology, we compared the total distance and speed obtained using StarDist-2D and TrackMate with those obtained by manual tracking. Manual tracking was performed by counting the number of times a stripe exited the field of view on the right side of the video. Each counted event corresponded to a displacement equivalent to the reference distance between two adjacent stripes (5 mm). Total distance was calculated as the number of counted stripes multiplied by 5 mm. Speed was calculated as the distance traveled per 20 s section; each recording consisted of 4 sections per snail.

### Statistical analysis

An ordinary least squares (OLS) regression model was used to assess the relationship between dry mass and shell mass. To evaluate relationship between body size parameters and heart rate, we used standard major axis (SMA) linear regression models implemented in the *R* package *lmodel2* which accounts for error in both dependent and independent variables. When comparing HR and HRV parameters on the induced behavioral states, we used the Student t-test for the data with a normal distribution (as determined by the Shapiro-Wilk test) and the Wilcoxon-signed-rank test when the data showed a non-normal distribution. For the relationship between locomotion and heart rate, we relied on linearized mixed models that accounted for individual variability as a random effect and body mass as covariable, using the library *lm4r*. The correlation coefficients and p-values were obtained using the *lmerTest* and *MuMIn* libraries, respectively. All figures were generated using the library *ggplot2* and all figures further modified or designed with *Adobe illustrator*® (v.29.5.1, 64-bit, Adobe Inc., 2025).

### Permits statement

No collecting permits were required for the collection or handling of snails due to the species’ status as an invasive species. Nevertheless, all procedures were conducted in accordance with ethical standards for the handling of animals, as established by Colombian legislation (Law 84 of 1989).

## Results

### Characterization of body size and correlations with average heart rate

We collected and measured body size heart activity from 27 snails in the immobilized and moving states. Individuals were excluded due to shell breakage (N = 2), experimental errors in laser alignment and snail position (N = 2), or excessive optocardiographic noise (N = 3). In total, 20 snails were successfully analyzed. We found Mass_T_ to vary between 1.37 g and 10.32 g (median±IQR = 3.48 ± 3.81 g). Dry mass varied between 0.08 g and 1.10 g (median±IQR = 0.28 ± 0.40 g) and shell mass varied between 0.09 g and 1.86 g (median±IQR = 0.38 ± 0.77 g). These variables were positively correlated (*β*_*1*_ = 1.60; R^2^ = 0.86, p < 0.001), indicating that investments in shells disproportionally increased with bigger metabolically active tissue (dry mass).

Then, we analyzed the correlation between average heart rate and the proxies of size: dry mass, shell mass and Mass_T_. We did not find heart rate to correlate with any parameter of body size independently of the retracted or moving state (S2 Table).

### Heart rate in retracted vs. moving states

**Fig 2:**
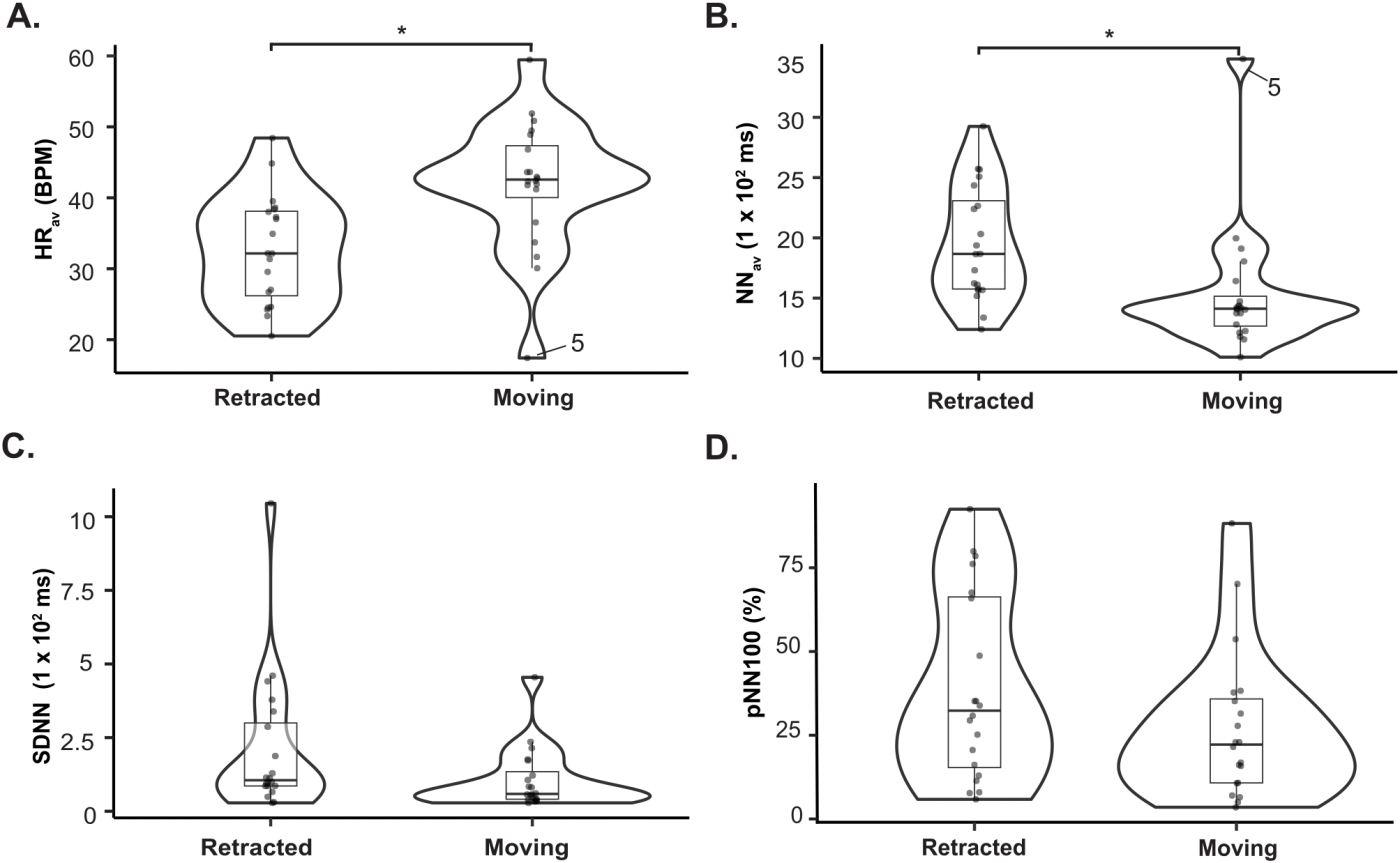
Heart rate and heart rate variability time-domain analysis in two induced-behavioral states (retracted and moving). **A.** Heart rate average (HR_av_) comparison between conditions. **B.** N-to-N intervals average (NN_av_). **C.** SDNN. **D.** pNN100. Significant p-values are indicated by asterisks (p < 0.005). Data distribution is shown with violin plots, while median and interquartile ranges (IQR) with embedded boxplots. Gray points represent the individuals measured in both states. Significant differences are marked with one asterisk (*). Individual 5 with an evident different pattern is shown for HR_av_ and NN_av_ comparison.

We found that heart rate significantly varied between retracted (mean±s.e.m = 32.7 ± 1.7 BPM) and moving states (mean±s.e.m = 42.0 ± 2.0 BPM); Paired Wilcoxon Signed-Rank test: 101, p < 0.0001). On average, snails increased a 1.31-fold their heart rate during the locomotion state compared to retraction (mean fold change±SD = 1.31 ± 0.28, n = 20; Fig 2A; Table 1). Accordingly, NN-intervals were significantly larger in the retracted state than in the moving state (Fig 2B, Table 1). Also, NN-intervals’ average had lower variation between snails in the moving state (Fig 2B, Table 1). Interestingly, in one snail (individual 5), heart rate when moving slightly decreased relative to the retracted state (HR_retracted_: 20.5 BPM, HR_moving_: 17.4 BPM; Fig 2A).

**Table 1:** Cardiac activity in experimentally induced retracted and locomotor states in snails.

Analysis	Variable	[mean±s.d.][Median-IQR]		Significance of change
		Retracted	Moving	
Heart rate	^HR_aver_ (BPM)	[32.6**±**7.6]	[42.0**±**9.1]	**P < 0.001
NN intervals	NN_aver_ (ms)	[1866.7-732.5]	[1412.3-248.0]	**P < 0.001
Time-domain	SDNN (ms)	[105.3-213.9]	[71.1-93.6]	P = 0.13
	pNN100 (%)	[30.5-50.9]	[17.3-25.1]	P = 0.11

We also analyzed the speed during 80 s recordings as a proxy for physical activity intensity. Only eight individuals could be included in this analysis due to failures in stripe detection during video processing and image analysis caused by insufficient lighting on the treadmill. The automated methodology for speed analysis using StarDist-2D and TrackMate was compared to manual tracking for validation, with differences in displacement of less than 5 mm and speed of less than 0.3 cm·min ⁻ ¹ (S3 Table). Snail speed ranged from 0.02 cm ⋅ min ⁻ ¹ to 12.3 cm ⋅ min ⁻ ¹ (median±IQR = 3.3 ± 3.9 cm ⋅ min ⁻ ¹). We found speed to correlate with instantaneous heart rate measured every five seconds. However, the effect size was small, predicting only a slight decrease in heart rate with increasing speed (instHR = 48.1–0.1*Speed+Snail+Mass_T_+error; p < 0.05, marginal-R² = 0.03; conditional-R² = 0.93). Moreover, body size and body composition did not significantly influence this relationship (p > 0.50).

### Heart rate variability changes by both retracted and moving individuals

Using a time-domain approach, we found that SDNN decreased by approximately 42% (median fold-change = 0.58, p = 0.13) from the retracted stated to the moving state (Fig 2C, Table 1). Similarly, pNN100 decreased by approximately 43% from the retracted stated to -moving state (median fold-change = 0.57, Fig 2D; Table 1, p = 0.11).

**Fig 3:**
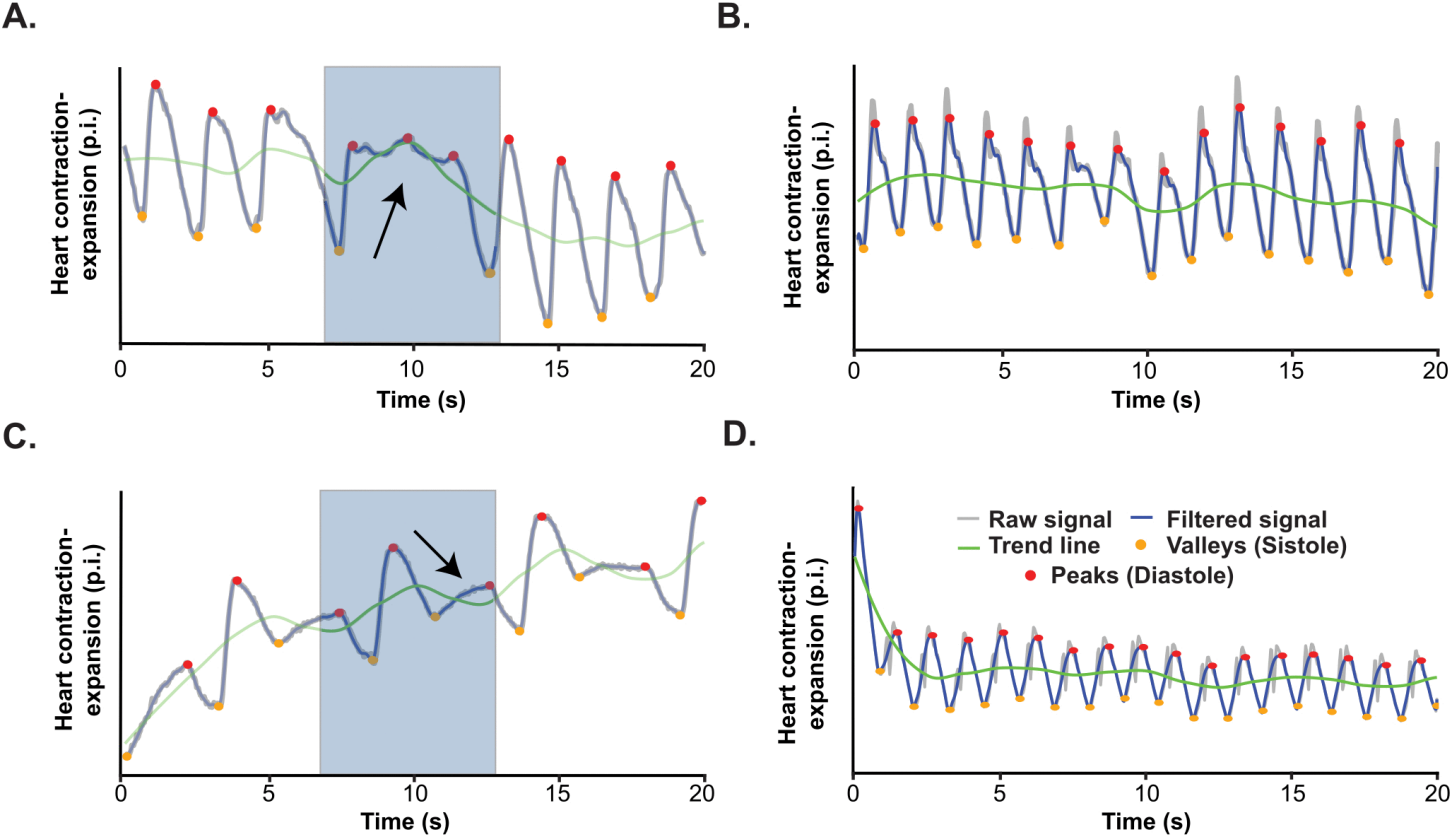
Optocardiograms in two experimentally induced behavioral states (retracted and moving). **A.** Example of a snail with a noticeable irregularity (black arrow) in the retracted state. **B.** Same snail as A. but in the moving state, where irregularities disappeared. **C.** Example of the snail with incomplete diastoles (Black arrow) in the retracted state. **D.** Same snail as C. in the moving state, where incomplete contractions disappeared.

In addition, we found five retracted individuals with evident irregular ventricular activity (Fig 3). We randomly selected a slice of 20 s from our optocardiograms derived from 80second-recordings to describe the irregularities. The irregularities from 4 snails were characterized by time differences and prolonged cardiac pauses during maximum diastole (Fig 3A, S1 Movie). Another individual failed to complete the maximum systole and, shortly afterward, it entered diastole (Fig 3C, S2 Movie). Even more impressive, all the irregularities from individuals disappeared during moving state (Fig 3B, D; S1 Movie, S2 Movie).

For Results with normal distribution (evaluated with Shapiro-Wilk test), Mean (X) and Standard deviation (s.d.) are reported. For non-normal data, median and Interquartile range (IQR) are shown. Statistical significance of change was assessed at the 95% confidence level. The Student’s t-test was used for comparison of normally distributed data, and the Wilcoxon signed-rank test for non-normal data. Normal data are indicated with one asterisk (^). Significant P-values are indicated with two asterisks (**).

## Discussion

Physiological adjustments are fundamental components of survival behaviors in animals. In terrestrial snails, the passive behaviors of body retraction and slow locomotion must impose unique challenges to the cardiovascular function. To understand the heart dynamics associated with retraction and locomotion in *C. aspersum*, we assessed HR and HRV during these two experimentally induced survival behaviors. We observed an increase in HR from the retracted to the moving state. We also found a trend toward reduced HRV and cardiac irregularities during locomotion. Moreover, locomotion intensity did not significantly influence HR. Interestingly, heart rate was independent of body size and body composition during these induced behavioral states. These findings may be explained by 1) Increased metabolic demands during locomotion, 2) Inhibitory and excitatory activity within neural pathways regulating cardiac function, 3) Differences in mechanical configuration of the circulatory system during experimentally induced behaviors, and 4) Compensation through other functional and anatomical strategies in cardiovascular system combined with the limitations of the experimental set up.

First, in our study, the assessed experimentally induced behaviors (i.e., retraction and movement) significantly influenced heart activity in *C. aspersum* (Fig 2A-D; Table 1). Most individuals exhibited an increased heart rate during movement and an associated decrease in NN intervals (Fig 2A, B; Table 1). During locomotion, organisms experience increased oxygen and energy demands, which are typically met through elevated cardiac output (CO) via increases in heart rate and/or stroke volume [6,32,33] Unsurprisingly, our snails as well as other invertebrates, including other mollusks, increase their heart rate during active states [34–36], positioning locomotion as one of the major determinants of cardiac dynamics. Nevertheless, we did not observe an increase in HR correlated with higher speed despite of increases of more than threefold. We found this to be surprising and suggest that the slow movement of individuals may be sufficiently covered by minimum increases in heart rate. Also, the contraction of the muscular foot may contribute as an auxiliary pump, complementing cardiac output [37]. Alternatively, the energy requirements may be primarily supplied by increases in stroke volume rather than higher heart rate as occurs in *Octopus vulgaris* during propulsion [6,38]. Nevertheless, the present findings should be interpreted with caution due to the limited sample size, and additional studies including a larger number of individuals will be required before general conclusions can be drawn for *C. aspersum*.

Second, cardioregulatory activity of the nervous system may be involved in the observed heartbeat fluctuations under different experimentally induced behavioral states. Across time-domain HRV metrics, we observed reduction in SDNN and PNN100 from the retracted to the moving state (Fig 2C-D, Table 1). Although invertebrates lack a vertebrate-like autonomic nervous system division, their neural pathways still have excitatory and inhibitory activity [16]. Some authors have referred to this system as autonomic-like due to its functional similarities with vertebrate “fight or flight” response [39–41]. In snails, heart function is under neurohumoral control, with both excitatory and inhibitory neurotransmitters modulating contractile activity in the auricle, ventricle, and pericardium [42]. Specifically, acetylcholine (ACh) reduces heart activity, while serotonin (5-HT) promotes excitation [43]. Moreover, in *Helix* species, specialized excitatory and inhibitory cardiac neurons have been identified and are thought to regulate heartbeat patterns [1]. Hence, during locomotion, neural pathways may underlie the decreases in HRV (and the associated HR increase) helping transport more O_2_ to the muscles involved in locomotion [7].

Thirdly, mechanical configurations of the circulatory system during experimentally induced behaviors may have impacted HRV observed here, by restraining the ventricular filling and heart contraction [37]. We observed evident irregularities associated with high HRV in snails under the retracted state (Fig 3). These anomalies may have raised from increased pericardial pressure, and insufficient ventricular filling that restrict contractility [37]. Elevated pericardial pressure may restrict auricular expansion, limit ventricular filling, and result in incomplete systoles [37,44]. Alternatively, diastolic pauses could reflect reduced venous return or mechanical impediment to ventricular contraction [45,46]. These disruptions may serve as energy-saving strategies under low metabolic demand [47,48] or just derive from biomechanical blood pressure limitations in open circulatory systems [44,49]. Supporting this, when the snails began moving, their cardiac irregularities resolved, suggesting relief of pericardial constraints during the locomotor activity. Similarly, irregularities have been reported in *Helix pomatia, Achatina fulica* [45,50–52] and in other invertebrate taxa [53,54], but without clear underlying causes.

An exception to the found patterns in HR and HRV across the experimentally induced behavioral states assessed was *Individual 5*, which exhibited the lowest heart rates in both behavioral states (Fig 2A). Despite being under the same condition as others, it exhibited a slight decrease in heart rate while active, accompanied by larger NN intervals (Fig 2A, B). Whether this finding suggests compensation through stroke volume rather than heart rate (see above) [6] requires further investigation. Releasing the pressure from the retracted state could enhance venous return, increasing vessels pressure caused by hemolymph circulation and increasing ventricular filling without an increase in heart rate. [44,55]. Also, when we excluded *Individual 5* from our analysis, we found significant differences on the SDNN and on pNN100 between the two behaviors (p < 0.05), supporting the idea that heart rate variability is influenced by the behavioral state.

Fourth, our results showed that heart rate during the evaluated induced behavioral states was independent of body size or composition (S2 Table). In principle, increases in metabolically active tissues (i.e., soft tissue, here measured with dry mass) and metabolically inactive tissues (i.e., shell, here measured with shell mass) should impose greater metabolic demands [17,56], requiring adjustment in cardiac function and anatomy to ensure adequate oxygen delivery and nutrient distribution in larger individuals [57,58]. In contrast to our findings, previous studies in invertebrates, including gastropod mollusks have often reported significant negative relationships between body size and heart rate [59,60], though exceptions exist in some taxa [55,61]. Specifically, other attempts in *C. aspersum* reported a negative relationship between heart rate and body mass [62], but they highlighted substantial variability related to latitude. This divergence suggests that our studied individuals may have physiological (e.g., higher stroke volume) or anatomical adaptations (greater heart size) that support the cardiovascular function to meet the increased metabolic demands associated with growth [63,64]. Moreover, our individuals were collected and tested at high elevation; whether this produces particular adaptations requires further investigation.

Finally, we propose that the evaluated physiological states and experimental set up could have masked the expected effect of body size on heart rate. If activity levels modulate heart rate more strongly than morphological traits, the influence of body size may only become detectable under standardized resting conditions. Moreover, our treadmill setup for the locomotion state may have reduced the metabolic cost associated with carrying the shell, as snails were held with the clamps, releasing the shell load. This mechanical unloading likely reduced the energy demands normally imposed by higher shell and body weight during locomotion [65]. Thus, the reduction in load may have attenuated the cardiovascular adjustments, reducing the effect of body size in heart rate during this behavioral state. In addition, our results do not allow the establishment of species-wide reference values for heart function under natural behavioral states, as these states were experimentally induced using a novel methodology and the sample size was limited. Therefore, our approach is restricted to comparing cardiac responses between retracted and locomotor states under controlled experimental conditions, serving as a proxy for physiological adjustment rather than as a basis for establishing reference values under natural conditions.

The cardiac activity of *Cornu aspersum* may be influenced by its behavioral state. During both retraction and locomotion, the observed changes in heart dynamics could be associated with differences in metabolic demand, mechanical loading and possibly neural modulation. However, these mechanisms were not directly tested in the present study. Therefore, our results should be interpreted as descriptive patterns consistent with these possibilities rather than as evidence of causal relationships. Hence, our results suggest that cardiovascular function in land snails may be behavior-dependent and could be influenced by the mechanical characteristics of their circulatory system, although this interpretation requires further experimental support. More broadly, our observations are consistent with the idea that physiological responses can change dynamically with behavior under controlled experimental conditions in terrestrial snails. Future research integrating neural recordings, metabolic measurements, and biomechanical analyses and higher sample size will help to determine the extent to which cardiac modulation during behavior is driven by neural control, mechanical constraints, or other physiological factors, thereby improving our understanding of the functional limitations and adaptive responses of different terrestrial mollusks to face changing environments.

## Supporting information

S1 File Raw dataset. It includes time and pixel intensity from ventricular contractions (Valleys); heart rate and heart rate variability parameters across experimentally induced behavioral states and body size; raw treadmill position data over time; instantaneous heart rate and average speed measured at 5-second intervals.
(XLSX)

S1 Table Cardiac parameters used for data analysis. *NN: Interval between ventricular contractions; **HR_inst_: Instantaneous heart rate; ***NN > 10 ms: Number of consecutive NN pairs differing by more than 100 ms; NN_T_: total number of NN pairs. HR, NN intervals, time-domain and frequency-domain metrics were obtained with R package *RHRV*. HR_5s_ and pNN100 were obtained independently using R. Definitions follow the standards for HRV analysis [29].
(DOCX)

S2 Table Correlations between body variables and heart rate during experimentally induced behavioral states (retracted and free-moving). SMA correlations were applied to analyze relationships between body variables and Heart rate. N = 20 snails. Coefficients of determination (R^2^) are shown. *β*_*1*_ = Slope. Statistical significance of correlations was assessed at the 95% confidence level.
(DOCX)

S3 Table Comparison between manual stripe movement tracking and StarDist-2D-TrackMate automated tracking. Total distance was calculated manually as the sum of the distances traveled by stripes across the four sections (4 sections per snail treadmill recording). Speed was calculated as the average speed across the four sections. Since manual detection is based on stripe counts, the estimated error in distance is 5 mm. Absolute differences in distance and speed between manual and automated detection are shown.
(DOCX)

S1 Movie First type of cardiac irregularities observed in four individuals. On the left, a representative individual shows pronounced variability in beat-to-beat intervals and prolonged cardiac pauses during maximal diastole in the retracted state. On the right, all cardiac irregularities disappear in the free-moving state in the same individual.
(MP4)

S2 Movie Second type of cardiac irregularities observed in one individual. On the left, the individual fails to complete maximal systoles during the retracted state. On the right, all cardiac irregularities disappear in the free-moving state in the same individual.
(MP4)
