# Supplementary material for: Cardiovascular adjustments during experimentally induced retraction and locomotion in the invasive terrestrial snail Cornu aspersum (Müller, 1774)
Source: PLoS One. 2026 Jul 31;21(7):e0354962. doi: 10.1371/journal.pone.0354962 (PMC13426994; doi:10.1371/journal.pone.0354962)
Supplement: S1 Table — *NN: Interval between ventricular contractions; **HRinst: Instantaneous heart rate; ***NN > 10 ms: Number of consecutive NN pairs differing by more than 100 ms; NNT: total number of NN pairs. HR, NN intervals, time-domain and frequency-domain metrics were obtained with R package RHRV. HR5s and pNN100 were obtained independently using R. Definitions follow the standards for HRV analysis [29]. (DOCX) [file pone.0354962.s002.docx]

**S1 Table. Cardiac parameters used for data analysis**.

| **Metric** | **Definition** | **Domain** | **Formula** |
| --- | --- | --- | --- |
| HR_av_  (BPM) | Average of the number of heartbeats in 60 seconds | Cardiac parameter | $* \frac{60 s}{\bar{NN}_{average}}$ (1). |
| HR_5s_  (BPM) | Average of the instantaneous heart rate for each 5 seconds | Cardiac parameter | $** \frac{1}{N}\sum{HR}_{inst}$ (2). |
| NN_av_ (ms) | Average of the intervals between consecutive ventricular contractions (beat-to beat). | Cardiac parameter | $***\frac{\sum_{i=1}^{N-1} NN}{{NN}_{T}}$ (3). |
| pNN100  (%) | Percentage of cardiac intervals that differ in more than 100 ms | Time | $\frac{NN>100 ms}{{NN}_{T}}\times100$ (4). |
| SDNN  (ms) | Standard deviation of NN intervals | Time | $\sqrt{\frac{1}{N-1}\sum_{i=1}^{N} {({NN}_{i+1}-{NN}_{mean})}^{2}}$ (5). |

*NN: Interval between ventricular contractions; **HR_inst_: Instantaneous heart rate; ***NN>10 ms: Number of consecutive NN pairs differing by more than 100 ms; NN_T_: total number of NN pairs. HR, NN intervals, time-domain and frequency-domain metrics were obtained with R package *RHRV*. HR_5s_ and pNN100 were obtained independently using R. Definitions follow the standards for HRV analysis [29].
