# Supplementary material for: Cardiovascular adjustments during experimentally induced retraction and locomotion in the invasive terrestrial snail Cornu aspersum (Müller, 1774)
Source: PLoS One. 2026 Jul 31;21(7):e0354962. doi: 10.1371/journal.pone.0354962 (PMC13426994; doi:10.1371/journal.pone.0354962)
Supplement: S2 Table — SMA correlations were applied to analyze relationships between body variables and Heart rate. N = 20 snails. Coefficients of determination (R2) are shown. β1 = Slope. Statistical significance of correlations was assessed at the 95% confidence level. (DOCX) [file pone.0354962.s003.docx]

**S2 Table. Correlations between body variables and heart rate during behavioral states (retracted and free-moving)**.

| **Variable** | **Factor** | **State** | ***β_1_*** | ***R^2^*** | ***P*** |
| --- | --- | --- | --- | --- | --- |
| **Heart Rate (BPM)** | **Mass_T_ (g)** | **Retracted** | **3.21** | **0.02** | **0.61** |
|  |  | **Free-moving** | **-2.69** | **0.01** | **0.74** |
|  | **Dry mass (g)** | **Retracted** | **-22.61** | **0.01** | **0.68** |
|  |  | **Free-moving** | **27.03** | **0.03** | **0.51** |
|  | **Shell mass (g)** | **Retracted** | **-13.12** | **0.03** | **0.48** |
|  |  | **Free-moving** | **15.68** | **0.00** | **0.82** |
|  | **Shell height (mm)** | **Retracted** | **-1.53** | **0.01** | **0.68** |
|  |  | **Free-moving** | **1.83** | **0.01** | **0.75** |
|  | **Shell diameter (mm)** | **Retracted** | **-2.25** | **0.01** | **0.78** |
|  |  | **Free-moving** | **2.69** | **0.01** | **0.65** |

SMA correlations were applied to analyze relationships between body variables and Heart rate. N=20 snails. Coefficients of determination (R^2^) are shown. *β_1_*= Slope. Statistical significance of correlations was assessed at the 95% confidence level.
