# Supplementary material for: Cardiovascular adjustments during experimentally induced retraction and locomotion in the invasive terrestrial snail Cornu aspersum (Müller, 1774)
Source: PLoS One. 2026 Jul 31;21(7):e0354962. doi: 10.1371/journal.pone.0354962 (PMC13426994; doi:10.1371/journal.pone.0354962)
Supplement: S3 Table — Total distance was calculated manually as the sum of the distances traveled by stripes across the four sections (4 sections per snail treadmill recording). Speed was calculated as the average speed across the four sections. Since manual detection is based on stripe counts, the estimated error in distance is 5 mm. Absolute differences in distance and speed between manual and automated detection are shown. (DOCX) [file pone.0354962.s004.docx]

**S3 Table. Comparison between manual stripe movement tracking and StarDist-2D-TrackMate automated tracking.**

| Snail | Manual | | Stardist-Trackmate | | Difference (Abs Δ) | |
| --- | --- | --- | --- | --- | --- | --- |
|  | Distance (mm) | Speed (cm⋅min⁻¹) | Distance (mm) | Speed (cm⋅min⁻¹) | Distance (Δ mm) | Speed (Δ cm⋅min⁻¹) |
| 19 | 80 | 6 | 79.92 | 6.09 | 0.08 | 0.09 |
| 20 | 30 | 2.3 | 32.24 | 2.43 | 2.24 | 0.13 |
| 21 | 55 | 4.1 | 55.25 | 4.25 | 0.25 | 0.15 |
| 22 | 5 | 0.4 | 6.15 | 0.67 | 1.15 | 0.27 |
| 25 | 35 | 2.6 | 31.09 | 2.57 | 3.91 | 0.03 |
| 26 | 110 | 8.3 | 110.34 | 8.38 | 0.34 | 0.08 |
| 30 | 40 | 3 | 43.67 | 3.29 | 3.67 | 0.29 |
| 32 | 45 | 3.4 | 45.47 | 3.44 | 0.47 | 0.04 |

Total distance was calculated manually as the sum of the distances traveled by stripes across the four sections (4 sections per snail treadmill recording). Speed was calculated as the average speed across the four sections. Since manual detection is based on stripe counts, the estimated error in distance is 5 mm. Absolute differences in distance and speed between manual and automated detection are shown.
